# Compliance and Quality of Endoscopic Tattooing in Colorectal Cancer: A Retrospective Audit

**DOI:** 10.7759/cureus.100800

**Published:** 2026-01-05

**Authors:** Bilal Ahmad, Nikita Nighoskar, Ghita Himmi, Michael Lim, Mohamed Amin, Kareem Attia

**Affiliations:** 1 General Surgery, York and Scarborough Teaching Hospitals NHS Foundation Trust, York, GBR; 2 Colorectal Surgery, York and Scarborough Teaching Hospitals NHS Foundation Trust, York, GBR; 3 Speciality Registrar General Surgery, York and Scarborough Teaching Hospitals NHS Foundation Trust, York, GBR

**Keywords:** audit, colon cancer, colonoscopy, colorectal cancer, compliance, endoscopic tattooing, lesion localisation

## Abstract

Background

Colonoscopy is integral to the diagnosis and staging of colorectal cancer. Endoscopic tattooing enables accurate intraoperative localisation of malignant lesions, particularly when lesions are not visible on cross-sectional imaging. Despite established local guidelines, variability in tattooing practice and documentation persists, potentially affecting surgical planning and outcomes.

Objectives

This study aims to assess compliance with local endoscopic tattooing guidelines for colorectal cancer and evaluate the quality and consistency of current practice.

Methodology

A retrospective audit was conducted over a 12-month period (February 2019 to February 2020). Data were collected using multidisciplinary team (MDT) lists and extracted from colonoscopy reports, operative notes, and imaging records for 231 patients with colorectal cancer. Data were entered into a password-protected Microsoft Excel (Microsoft Corporation, Redmond, Washington) file and cross-checked for completeness and accuracy. Statistical analysis was performed using IBM SPSS Statistics for Windows, Version 29 (Released 2023; IBM Corp., Armonk, New York), and results were summarised using descriptive statistics and presented in tabular format. Findings were assessed against local trust guidelines and interpreted in the context of relevant literature.

Results

Of 142 lesions where tattooing was mandatory, only 102 (72%) were tattooed at the index colonoscopy. Fifty-seven lesions (40.1%) were not visible on CT imaging. Among non-tattooed lesions, 16 were CT-invisible; however, only nine underwent repeat endoscopic tattooing prior to surgery. Documentation quality was suboptimal, with adequate tattooing details recorded in only 41.4% of cases. These findings indicate inconsistent adherence to recommended practices, particularly in cases where accurate lesion localisation is most critical.

Conclusion

Compliance with local endoscopic tattooing guidelines was suboptimal, with significant deficiencies in both implementation and documentation. Inadequate tattooing and poor recording may hinder intraoperative localisation, particularly for CT-invisible tumours, potentially affecting surgical efficiency and outcomes. Targeted endoscopist education, standardised documentation proformas, and system-level interventions are required to improve compliance. These findings suggest that inadequate tattooing and poor documentation are most prevalent in lesions not visible on CT, where accurate endoscopic localisation is crucial for surgical planning. A repeat audit is recommended to evaluate the effectiveness of these measures and ensure sustained improvement in clinical practice.

## Introduction

Accurate localisation of colorectal lesions identified at colonoscopy is essential to ensure oncologically appropriate resections, particularly during laparoscopic surgery, where lesions may be impalpable. Inadequate localisation can lead to resection of an incorrect bowel segment, prolonged operative time, conversion to laparotomy, or unnecessary stoma formation, all of which increase perioperative morbidity, negatively impact patient quality of life, and place additional demands on NHS resources [1-4].

Endoscopic tattooing is widely used to facilitate the reliable preoperative localisation of colorectal lesions. First described by Ponsky and King in 1975, the technique involves submucosal injection of permanent carbon-based ink, typically following a saline lift to confirm correct submucosal placement and reduce the risk of transmural injection or peritoneal contamination [5]. Recommended practice includes placement of multiple injections distal to the lesion, ideally distributed circumferentially in three to four quadrants, to optimise intraoperative visibility while preserving surgical planes and avoiding inflammatory distortion [6]. Endoscopic tattooing has been shown to be superior to alternative localisation methods, such as endoscopic clip placement, particularly for early-stage or non-palpable tumours [5,7].

Recognising its clinical importance, national guidance has been developed to standardise tattooing practice. The Joint Advisory Group on Gastrointestinal Endoscopy (JAG), the British Society of Gastroenterology (BSG), and the Association of Coloproctology of Great Britain and Ireland (ACPGBI) recommend that all lesions ≥20 mm or suspicious for malignancy, located between the caecum and rectum, should be tattooed in accordance with local protocols [8]. Evidence suggests that inadequate tattoo volume compromises intraoperative visibility; injections of less than 1 mL may be undetectable in a significant proportion of cases, whereas volumes of 1-1.5 mL achieve visibility in up to 98.9% of surgeries without compromising surgical planes [6].

Despite clear guidance and a robust evidence base, variability in real-world adherence to recommended tattooing techniques and documentation standards persists. Inconsistent application of tattooing protocols and inadequate documentation may undermine effective surgical localisation, particularly for lesions that are not visible on preoperative imaging.

The aim of this study was to evaluate adherence to local endoscopic tattooing guidelines for colorectal cancer, assess the quality and documentation of tattooing practice, and identify areas for improvement in preoperative lesion localisation.

## Materials and methods

Study design

Data were collected retrospectively from colonoscopy reports over a 12-month period (February 2019 to February 2020) using multidisciplinary team (MDT) lists. A total of 300 patients with colorectal cancer were initially identified. Exclusion criteria were applied, resulting in 231 patients eligible for analysis.

Exclusion criteria

Patients were excluded if they had not undergone surgery due to metastatic disease or death prior to the procedure, if they presented acutely with conditions such as bowel obstruction and were therefore unable to undergo colonoscopy, or if their colonoscopy or surgical procedure was performed at another Trust with reports that were inaccessible for review.

Inclusion criteria

The audit included adult patients (≥18 years) with a confirmed diagnosis of colorectal cancer who underwent surgical resection at the trust and had complete and adequately documented colonoscopy and operative reports available for review.

Data collection

A structured database was created to record patient demographics (age, gender), endoscopist specialty, and procedural details, including lesion characteristics (location, lesion type, such as mass, polyp, or stricture), distance from the anal verge, scope traversability, and whether a tattoo was placed. Operative reports were reviewed to record surgery dates and assess whether the tattoo and lesion were visualised intraoperatively.

Tattooing procedure and quality assessment

**Figure 1:**
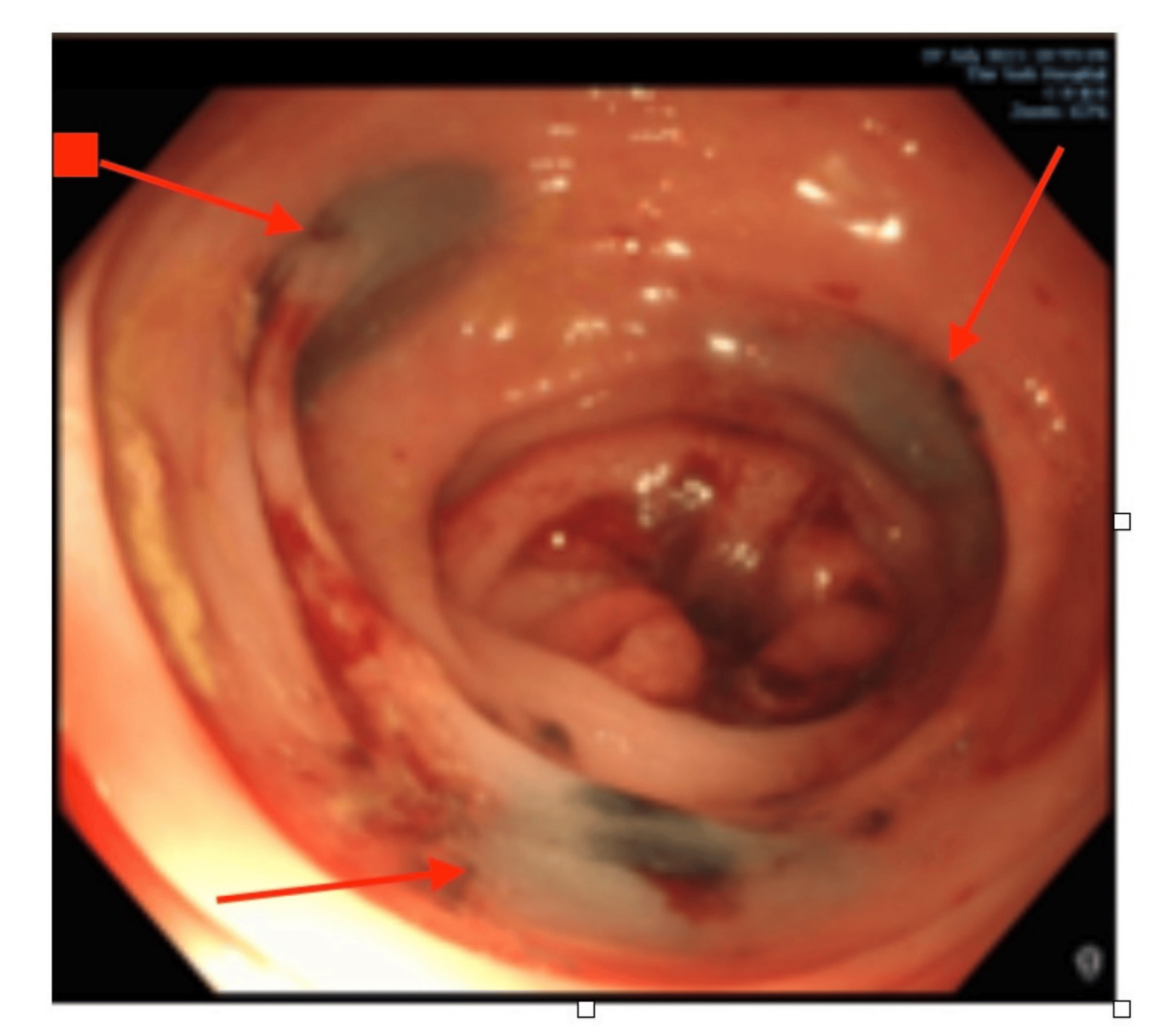
Photograph taken during a colonoscopy of a patient at York Hospital. The image shows a good-quality tattoo with three distinct blebs, 120 degrees apart, within the submucosa, and an appropriate amount of ink.

The use of tattooing to localise lesions during the index colonoscopy was recorded. Tattooing quality was evaluated using predefined criteria adapted from established practice at St Mark's Hospital, a recognised tertiary referral centre for colorectal disease. Specifically, adequate-quality tattooing was defined as documentation of (1) the use of permanent carbon-based ink, (2) placement distal to the lesion, (3) circumferential or multi-quadrant injection, and (4) a clear description of the tattoo location in relation to anatomical landmarks. Tattooing was classified as suboptimal if one or more of these elements were absent or inadequately documented. Tattooing was considered mandatory for all lesions except those located in the rectum, anus, and caecum. Tattoo quality was assessed on a 0-3 scale based on three criteria, each scoring one point. A distal tattoo was required to be placed close to, but not involving, the lesion; three separate blebs were injected at approximately 120-degree intervals within the submucosa around the bowel circumference (see Figure 1 for an example); and the tattoo needed to be easily detectable during surgery. A total score of 3 indicated high quality, scores of 1-2 indicated moderate quality, and a score of 0 indicated poor quality, in accordance with recommended guidelines.

Computerised tomography (CT) scan

Following histological confirmation of malignancy, patients underwent CT scanning for staging. Lesions were assessed for visibility on CT and for concordance with initial colonoscopy findings.

Surgical assessment and tattoo localisation

Surgical candidacy was determined by the colorectal MDT, with laparoscopic resection as the default approach. Tattoo visibility and lesion localisation were assessed intraoperatively, with any difficulties documented in the database.

Statistical analysis

Data were entered into a password-protected Microsoft Excel (Microsoft Corporation, Redmond, Washington) file and cross-checked for completeness and accuracy. Analyses were performed using IBM SPSS Statistics for Windows, Version 29 (Released 2023; IBM Corp., Armonk, New York). Categorical variables, such as tattoo placement, CT visibility, lesion type, and documentation quality, were summarised using frequencies and percentages, while continuous variables, such as patient age and distance from the anal verge, were reported as mean ± standard deviation or median (interquartile range), depending on distribution. Ordinal variables, including tattoo quality scores, were summarised using medians and interquartile ranges.

Comparisons between groups (e.g., tattooed vs. non-tattooed lesions, CT-visible vs. CT-invisible lesions) were conducted using appropriate tests for variable type, and subgroup differences were assessed where relevant. Cases with missing data for a variable were excluded from analyses involving that variable. The statistical analyses were primarily descriptive and intended to characterise local tattooing practices, CT visibility, and documentation quality. Results are presented in tables with counts, percentages, and summary statistics to provide a clear overview of practice patterns and adherence to local guidelines.

## Results

Study population

After applying the exclusion criteria, 231 patients remained for analysis. The median age was 71 years (IQR 55-87), with 175 male patients. Of the 231 patients, 142 lesions were located between the rectum and caecum, where tattooing was considered mandatory. Table 1 summarises the anatomical distribution of lesions (N-numbers) across colonic segments, including the ascending colon, hepatic flexure, transverse colon, splenic flexure, descending colon, and sigmoid colon.

**Table 1:** Distribution of lesions in colonic segments and lesions tattooed at the initial colonoscopy across different colonic locations. Numbers and percentages are shown.

Location of the Colon	Tattoo on Initial Scope N=102 (%)	Not Tattooed on Initial Scope N=40 (%)
Ascending (N=31)	18 (58%)	13 (42%)
Hepatic (N=13)	12 (92%)	1 (8%)
Transverse (N=11)	8 (73%)	3 (27%)
Splenic (N=7)	6 (86%)	1 (14%)
Descending (N=5)	5 (100%)	0 (0%)
Sigmoid (N=75)	53 (71%)	22 (29%)

Ethical approval

As a retrospective audit, formal ethical approval was not required according to local institutional policy.

Performance of the tattooing procedure

Tattooing was performed in 102 out of 142 lesions (72%) during the initial colonoscopy. Tattooing rates were lower among nurse endoscopists compared to gastroenterologists and general surgeons (8.8% vs. 31.4% vs. 59.8%), but this difference was not statistically significant (p=0.131). Tattooing frequency was similar between small polyp cancers and large mass lesions (84% vs. 74%, p=0.49). Among the 40 lesions not tattooed initially, only nine (22.5%) underwent a follow-up colonoscopy for tattoo placement. Tattoo use was also examined by lesion location within the colon to identify variations. Full details are shown in Table 1.

Assessment of tattoo quality

Including follow-up scopes, 111 lesions were tattooed during index or subsequent colonoscopy. However, 54 patients (49%) lacked sufficient descriptive or photographic documentation to fully assess tattoo quality. Specifically, 85% had no information on the number of tattoos, 44% lacked tattoo location details, and 30% had no documentation of tattoo visibility at surgery; these cases were excluded from quality scoring. Among the remaining 57 patients, 46 (80.7%) had tattoos graded as high quality according to the predefined criteria. The quality scoring system, even though it is not a published or standardised system from St Mark's Hospital, JAG, or ACPGBI, is a predefined scoring system used in this study to grade tattoo visibility, clarity, placement, and adequacy for surgical localisation [9]. Table 2 provides further details on tattoo quality among the 57 evaluable cases. It shows the distribution of quality scores (0-3), with the majority of tattoos graded as high quality (score 3).

**Table 2:** Distribution of tattoo quality scores (0–3) among 57 evaluable cases. Numbers and percentages of cases are shown for poor quality (score 0), moderate quality (scores 1–2), and high quality (score 3).

Quality Score	N (%)
0	1 (1.8%)
1	0 (0%)
2	10 (17.5%)
3	46 (80.7%)
Total	57 (100%)

Lesion visibility and localisation challenges

Among lesions for which endoscopic tattooing was mandatory, 57 (40.1%) were not visible on preoperative CT imaging. Of the 40 lesions that were not tattooed at the index colonoscopy, 16 (40%) were also CT-invisible. A repeat colonoscopy for tattoo placement was performed in nine patients (56%), confirming lesion localisation consistent with the initial colonoscopy findings. Despite the absence of both tattooing and radiological visibility, seven patients (44%) underwent successful surgical resection. Minor discrepancies between colonoscopic and CT localisation were observed but did not influence surgical management.

These findings highlight that radiological imaging alone is often insufficient for reliable lesion localisation. Accurate endoscopic tattooing at the index colonoscopy remains essential to ensure precise localisation, reduce the need for repeat procedures, and support safe and effective surgical planning.

## Discussion

This study evaluated compliance with our NHS Trust policy on endoscopic tattooing in patients with colorectal cancer. Overall adherence to local protocol was suboptimal, with incomplete documentation and missed tattooing most frequently observed in anatomically complex regions such as the ascending and sigmoid colon. Preoperative imaging modalities, including CT colonography, were often unreliable for lesion localisation, and in some cases, surgical resections were performed successfully without tattoos, likely relying on intraoperative landmarks or on-table colonoscopy. While alternative localisation techniques, such as barium enema, mucosal clips, and intraoperative colonoscopy, have been described, endoscopic tattooing with India ink remains the most practical and reliable method for intraoperative identification of colorectal lesions, particularly for early, flat, or non-stenosing tumours [2,5,7,9-11].

Our findings align with reports from other UK centres demonstrating suboptimal compliance. St Mark's Hospital documented a 71% adherence rate, attributing poor compliance to technical challenges and inadequate documentation; their revised protocol now mandates distal tattooing for all lesions to ensure intraoperative visibility [11]. Similarly, Queen's Medical Centre in Nottingham recommends standardised submucosal injection of 1 mL India ink at multiple points around the lesion to optimise localisation [4]. These observations are consistent with national guidance from JAG and BSG, which advocate 100% compliance for eligible lesions, highlighting a persistent gap between guideline expectations and real-world practice [8].

Inadequate tattooing and incomplete documentation have direct clinical and surgical implications. Failure to accurately localise lesions can increase operative complexity, risk of extended resections, and reliance on intraoperative judgement, which may not be consistently reproducible. Limited visibility on CT imaging further underscores the importance of standardised endoscopic localisation. Suboptimal documentation also affects surgical planning, audit processes, and quality improvement initiatives. Contributing factors likely include workflow inefficiencies, procedural time pressures, staffing limitations, and variable endoscopist experience or training [2].

This audit provides a comprehensive evaluation of local practice, benchmarked against national standards and peer institutions, highlighting areas for quality improvement. Limitations include its retrospective design, reliance on existing documentation, and single-centre scope, which may limit generalisability. Overall, the findings emphasise the critical importance of performing guideline-compliant endoscopic tattooing with accurate documentation at the index colonoscopy. Standardisation of technique, structured training, and robust documentation protocols are essential to improve lesion localisation, support surgical planning, and ensure adherence to national quality standards.

Limitations

Our study had several limitations. The sample size was reduced from 300 to 231 patients after applying exclusion criteria and was further narrowed to 142 patients eligible for compulsory tattooing. Selection bias may have arisen during data collection, particularly in cases where documentation was incomplete or missing. Patients treated across multiple trusts were excluded due to a lack of access to surgical notes, which may have skewed the results. Additionally, there was heterogeneity in the quality and format of documentation across endoscopy and surgical reports, with inconsistencies requiring cross-referencing with MDT minutes, which were often lacking in detail. The scoring system used in this study is novel, and although the BSG, the National Bowel Cancer Screening Program, and other expert consensus documents recommend tattooing all lesions outside the rectum, anus, and caecum to aid surgical localisation, it may be subject to observer bias. Inter-operator consistency was not formally assessed, and the surgical visibility of the tattoo is influenced by intraoperative factors that may not solely reflect endoscopic technique.

Recommendations

We propose a threefold intervention strategy to improve compliance. First, local trust posters should be redesigned to be clearer and more visible in endoscopy rooms, summarising tattooing indications and techniques. Second, an electronic proforma should be implemented in the endoscopy documentation system, requiring endoscopists to record tattoo numbers and their positions relative to the lesion. Third, educational sessions should be delivered at Endoscopy User Group meetings, accompanied by feedback on audit findings. A re-audit is planned within 6-12 months to assess the impact of these interventions.

## Conclusions

In conclusion, all colonic lesions that appear to be malignant should be tattooed during endoscopy to improve surgical localisation. Targeted educational programmes and system-level interventions are planned to improve endoscopic tattooing practices and documentation. These measures aim to enhance surgical planning and patient safety. A re-audit will be conducted within 6-12 months to assess their impact.
